# Data that support the use of agro-industrial residues from orange peel and sugarcane bagasse for the production of carbonaceous structures and their application in the removal of metal ions

**DOI:** 10.1016/j.dib.2022.108410

**Published:** 2022-06-23

**Authors:** Á.I. Licona-Aguilar, A.M. Torres-Huerta, M.A. Domínguez-Crespo, D. Palma-Ramírez, E. Conde-Barajas, M.X.L. Negrete-Rodríguez, A.E. Rodríguez-Salazar, D.S. García-Zaleta

**Affiliations:** a Instituto Politécnico Nacional, CICATA-Altamira, CIAMS. km 14.5 carretera Tampico-Puerto Industrial Altamira, México; b Instituto Politécnico Nacional, UPIIH, Ciudad del conocimiento y la cultura. Carretera Pachuca-Actopan km. 1+500 San Agustin Tlaxiaca, Hidalgo C.P. 42162, México; c Instituto Politécnico Nacional, Centro Mexicano para la Producción más Limpia (CMPL), Av. Acueducto s/n, la Laguna Ticomán, México City C.P. 07340, México; d Departamento de Ingeniería Bioquímica, Departamento de Ingeniería Ambiental, TecNM/ IT de Celaya, Av. Tecnológico y A. García Cubas No. 600, Celaya, Guanajuato 38010, México; e Instituto Politécnico Nacional, CICATA Querétaro, Cerro Blanco 141, Col. Colinas del Cimatario, Santiago de Querétaro, Querétaro C.P. 76090. México; f Universidad Juárez Autónoma de Tabasco. Carretera Estatal Libre Villahermosa-Comalcalco. Km. 27 +000 s/n Ranchería Ribera Alta, Tabasco C.P. 86205, México

**Keywords:** Agroindustrial wastes, Orange peel, Sugarcane bagasse, Activate carbons, Carbon foams, Heavy metal ions removal

## Abstract

This document contains additional information for the production of activated carbons (AC) and carbon foams (CF) from agroindustrial wastes, orange peel (OP) and sugarcane bagasse (SCB). In particular, a set of data is presented for the characterization of carbonaceous structures (AC and CF) and their application in the removal of metallic ions contained in polluted waters. The adsorbent materials were obtained combining chemical and physical activation processes. Data presented here included characterization of AC and CF using dynamic light scattering (DLS), BET (Brunauer, Emmet and Teller) surface area analysis, Barrett-Joyner-Halenda (BJH) method to assess pore size distribution and zeta potential (ζ) to evaluate electrokinetic potential of carbonaceous structures. In addition, energy dispersive spectroscopy (SEM/EDS) to identify heavy metals on the surface of carbonaceous materials is shown and complementary adsorption capacity data for metal ion removal are presented in the paper. The data can be used as a reference to promote reuse of agroindustrial wastes and provide added value; particularly for the synthesis of carbonaceous structures applied to the water purification.

## Specifications Table

**Table:** 

Subject	Materials science, environmental science
Specific subject area	Activated carbon, carbon foams, heavy metals adsorption
Type of data	Tables, Figures, Text file
How the data were acquired	Derived from an agreement of the Mexican Government through laboratory experiments. Characterization was performed by dynamic light scattering (DLS) (Particle analyzer litesizer 500 Anton Paar); Adsorption- desorption analysis (Brunauer-Emmett-Teller, BET), Barrett-Joyner-Halenda (BJH) method using a Micromeritics Instrument TriStar II 3020. Scanning electron microscopy/energy dispersive X-ray spectroscopy (SEM/EDS) (JEOL JSM-6010LA microscope). Heavy metal concentrations in the synthetic water, before and after the CF purification process, were quantified by atomic absorption spectrometry (AAS) using a Perkin Elmer AAnalyst 400 equipment.
Data format	Filtered, fitted curves and analyzed data
Description of data collection	Chemical and physicochemical data of AC synthesized at two different temperatures (500 and 700 °C) were acquired as well as CF prepared with AC and polyvinyl alcohol, both used as carbon precursor and binder, respectively. The replica method was carried out using polyurethane foam as template. A 658 nm semiconductor laser (40 mW) was used for particle size determination. For Zeta potential (ζ), 5 mL of deionized water was added to 0.05 g of each sample (40 mW semiconductor laser of 658 wavelengths; Particle Analyzer, Anton Paar). Before measurements, the carbonaceous materials were dispersed in deionized water (Fisher Chemical, HPLC) and sonicated for 120 min to ensure adequate dispersion. Additionally, the effect of pH on ζ in carbonaceous samples, sintered at 700 °C, was analyzed. In this case, the analysis consisted of using 0.05 g of carbonaceous structures (AC or CF) that
	were added to 5 mL of deionized water; and the pH was adjusted from 2 to 8 using HCl or NaOH solutions (0.01 M). Samples were sonicated for 40 min. The solution was then transferred to a zeta potential cuvette for evaluation. Data were processed into figures using OriginPro 9.0. The surface area and pore volume were determined under the following conditions.1g of sample was used to acquire the nitrogen (N_2_) adsorption- desorption at 300 °C using a pressure of 6.58 × 10-5 Torr for 20 h. Experimental adsorption- desorption data were analyzed using the Langmuir, Freundlich and BET equations model and processed using OriginPro 9.0 software. An accelerating voltage of 20 kV at different magnifications was used to obtain SEM/EDS images. Cu and Pb concentrations were quantified through calibration curves using high purity standards (Sigma Aldrich, 1000 mg/L) and atomic absorption spectrometry (AAS).
Data source location	DLS spectra and Zeta potential were measured at the Instituto Politécnico Nacional IPN, CICATA – Altamira, C.P. 89600, Altamira, Tamaulipas, México, SEM/EDS images and AA spectroscopy were performed at the Escuela Superior de Ingeniería Química e Industrias Extractivas del Instituto Politécnico Nacional Ciudad de México, C.P. 07300 México, DF, México. BET analyzes were collected at Universidad Juárez Autónoma de Tabasco. C.P. 86040. Tabasco México.
Data accessibility	Repository name: Mendeley Data Data identification number: 10.17632/3gmbcyr5n7.2 Direct link to the dataset: https://data.mendeley.com/datasets/3gmbcyr5n7/2
Related research article	Á. I. Licona-Aguilar, A. M. Torres-Huerta, M. A. Domínguez-Crespo, D. Palma Ramírez, E. Conde-Barajas, M.X. L. Negrete- Rodríguez, A.E. Rodríguez-Salazar, D.S. García-Zaleta “Reutilization of waste biomass from sugarcane bagasse and orange peel to obtain carbon foams: applications in the metal ions removal”. Science of the total environment. Sci. Total Environ. 831 (2022) 154883. https://doi.org/10.1016/j.scitotenv.2022.154883[1]

## Value of the Data

• The data can be useful or used by researchers to compare the application of different biomasses to produce activated carbon or carbon foams for wastewater treatment.
• The data are valuable because it provides information on adsorption equilibrium; using two adsorbent materials: activated carbons and carbon foams synthesized from agro-industrial wastes (orange peel and sugarcane bagasse).
• Data show that agro-industrial wastes from orange peel and sugarcane bagasse can remove heavy metal ions and can be proposed for their used in treatment of water.
• BET data provide information on the effect of activation temperature on the pore size distribution and surface area of the material.
• Zeta potential (ζ) data give important insight on the effect of pH on the charge surface of material in relation to their capacity adsorption.
• The data from this research open up alternatives to propose and study orange peel and sugarcane bagasse to remove other types of contaminants.

## 1 Data Description

**Fig. 1:**
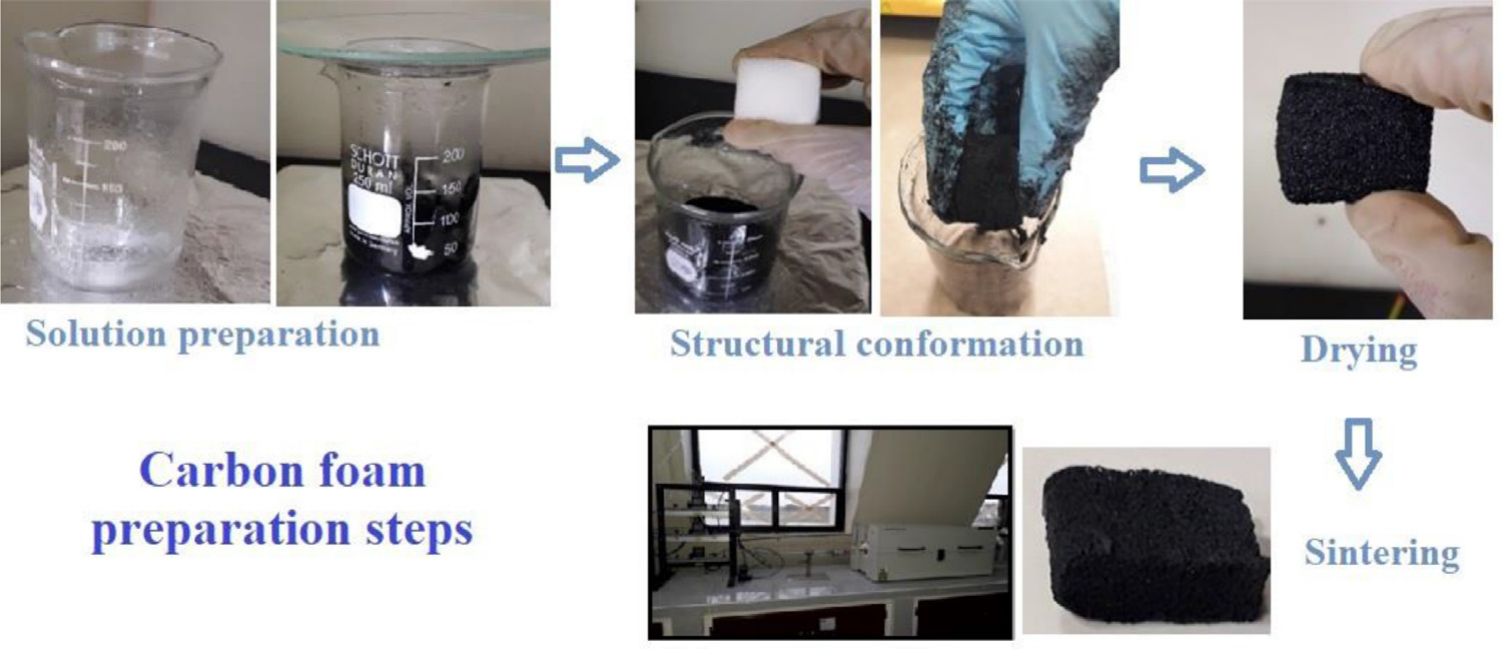
Carbon foam preparation.

**Fig. 2:**
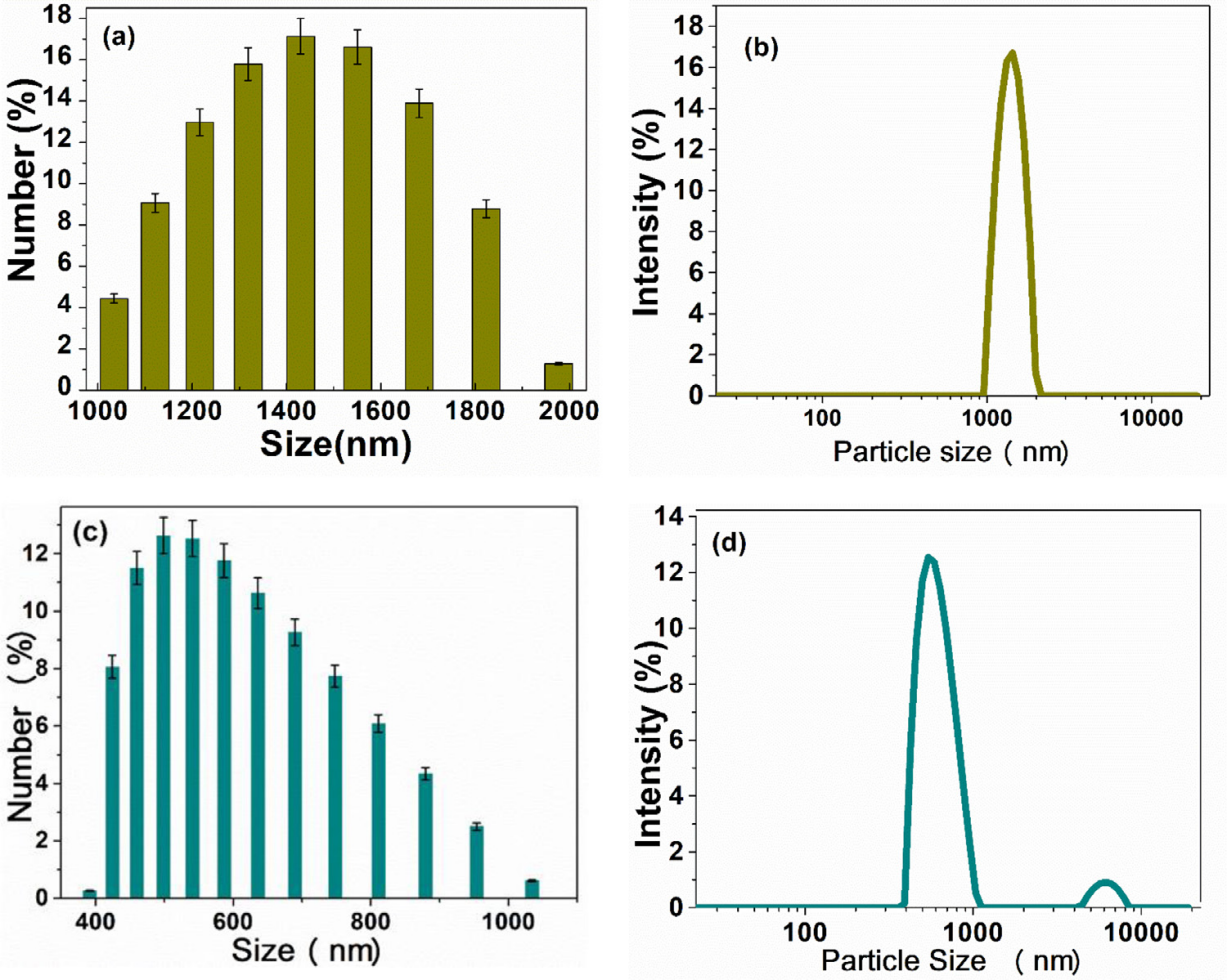
Particle Size distribution of AC from SCB at 700 °C (a-b), AC from OP at 700 °C (c, d).

**Fig. 3:**
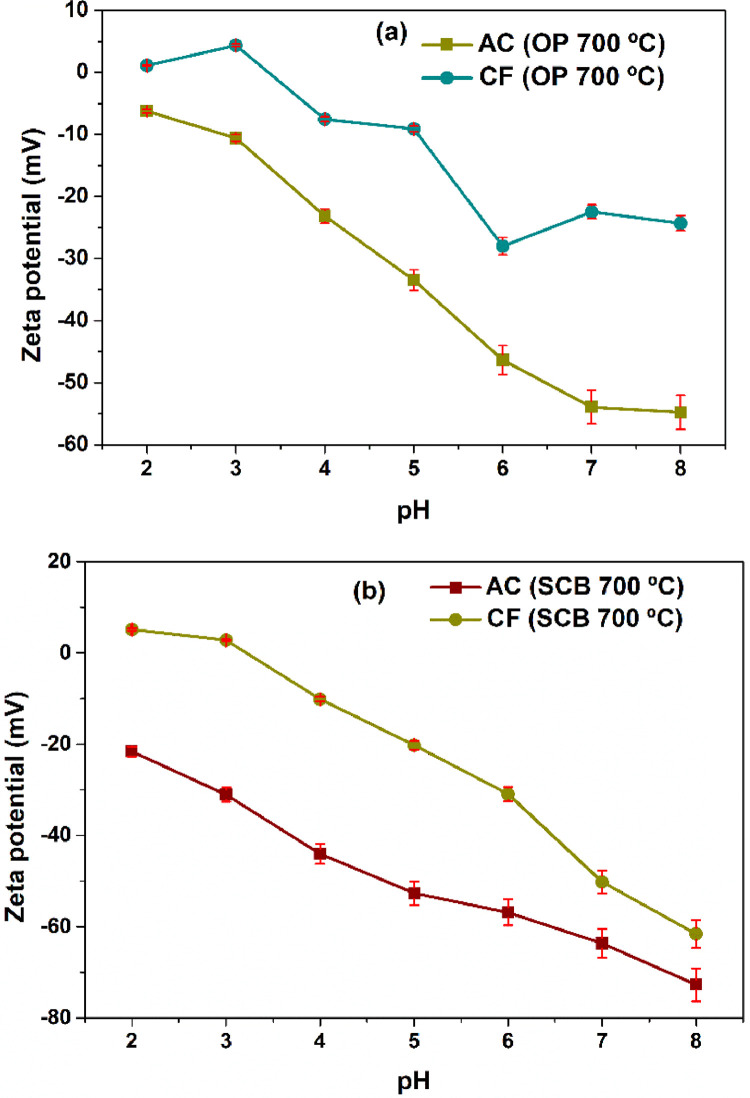
Zeta potential measurements of AC and CF form (a) OP and (b) SCB at 700 °C and different pH of the solution.

**Fig. 4:**
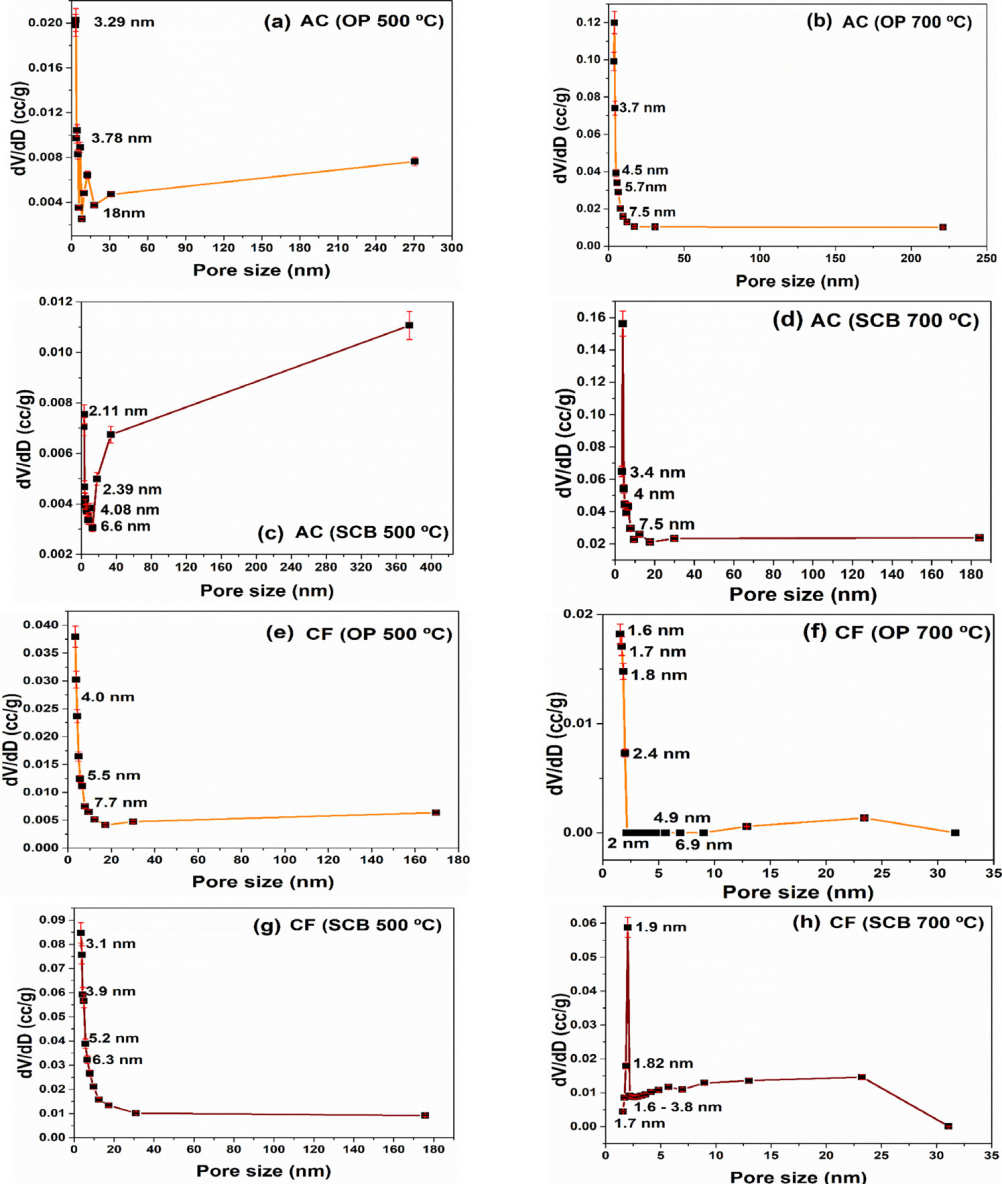
Pore size distribution of carbon materials, using different sintering temperatures during the synthesis.

**Fig. 5:**
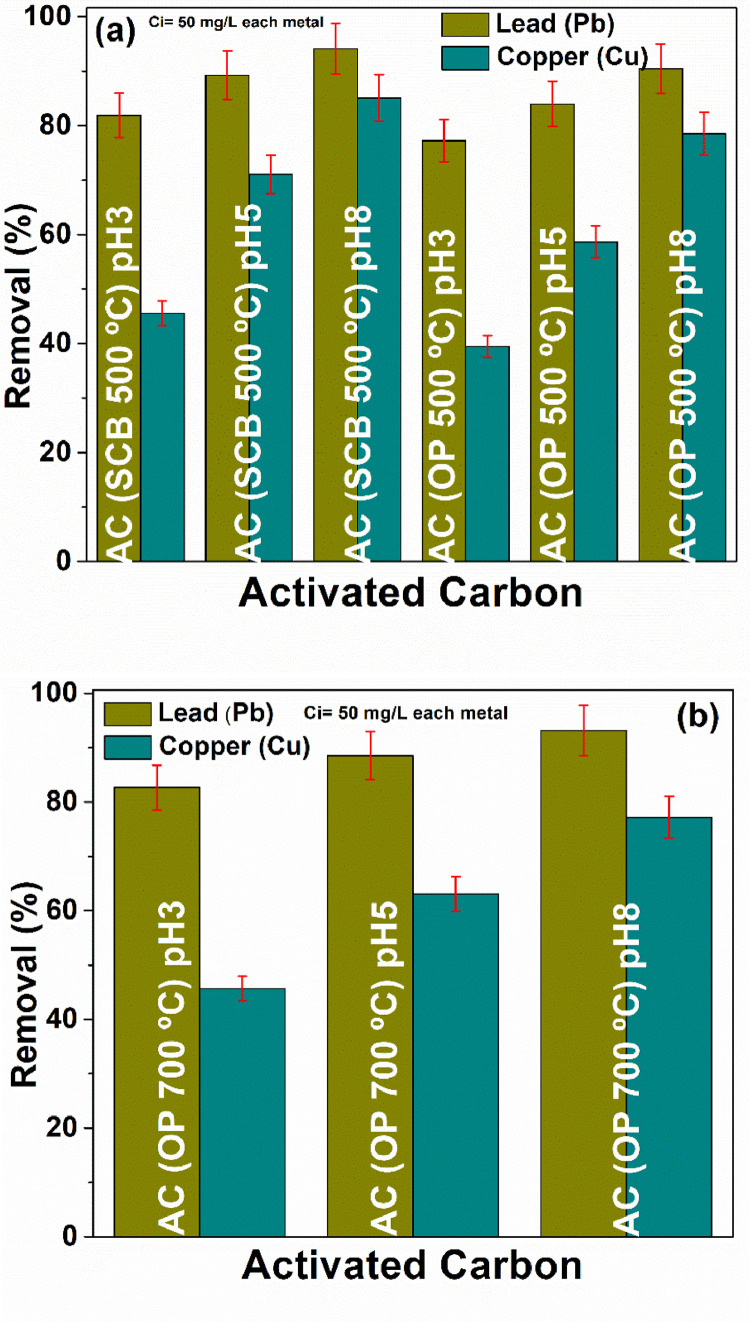
Adsorption capacity determination of AC (a) 500 °C and (b)700 ° (C).

Orange peel and sugarcane bagasse were used to obtain carbonaceous structures such as activated carbons and carbon foams for their application in the removal of heavy metal ions. The data set of this work shows information on the characterization and evaluation of these carbon materials that can be used as adsorbent materials, confirming recent reports [2], [3], [4], [5]. Fig. 1 shows the steps for the preparation of the carbon foam, using activated carbon from agro-industrial wastes as precursor and the replica method. Fig. 2 a–d shows dynamic light scattering (DLS) measurements to analyze the hydrodynamic radius of the samples synthesized from both agroindustrial wastes at 700 °C. Fig. 3 a–b shows the evolution of the Zeta potential (ζ) as a function of the pH solution that was used to predict adsorption capacity of the carbonaceous materials. Fig. 4 a–h shows the pore size distribution of the carbon structures of both agroindustrial wastes at different sintering temperatures. The quantification of the heavy metal removed (Pb or Cu) in each adsorbent material can be seen in the Fig. 5 a, b. The adsorption capacity results (Fig. 5) showed better lead removal efficiency. One possible explanation is the number of chemical groups such as hydroxyl, amine and carboxyl compounds; or minerals such as potassium, phosphorous that have a higher greater for the sorption of lead.

**Fig. 6:**
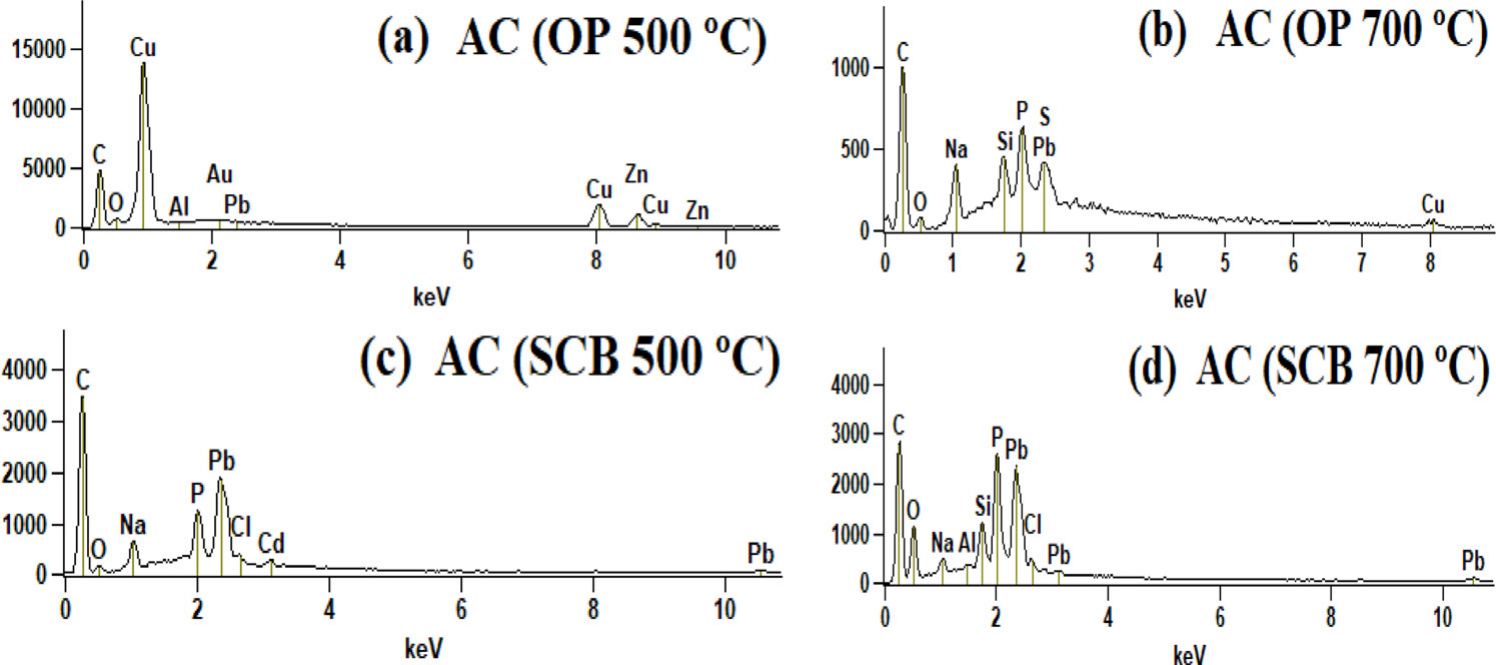
SEM/EDS analysis of activated carbons at different temperatures.

**Fig. 7:**
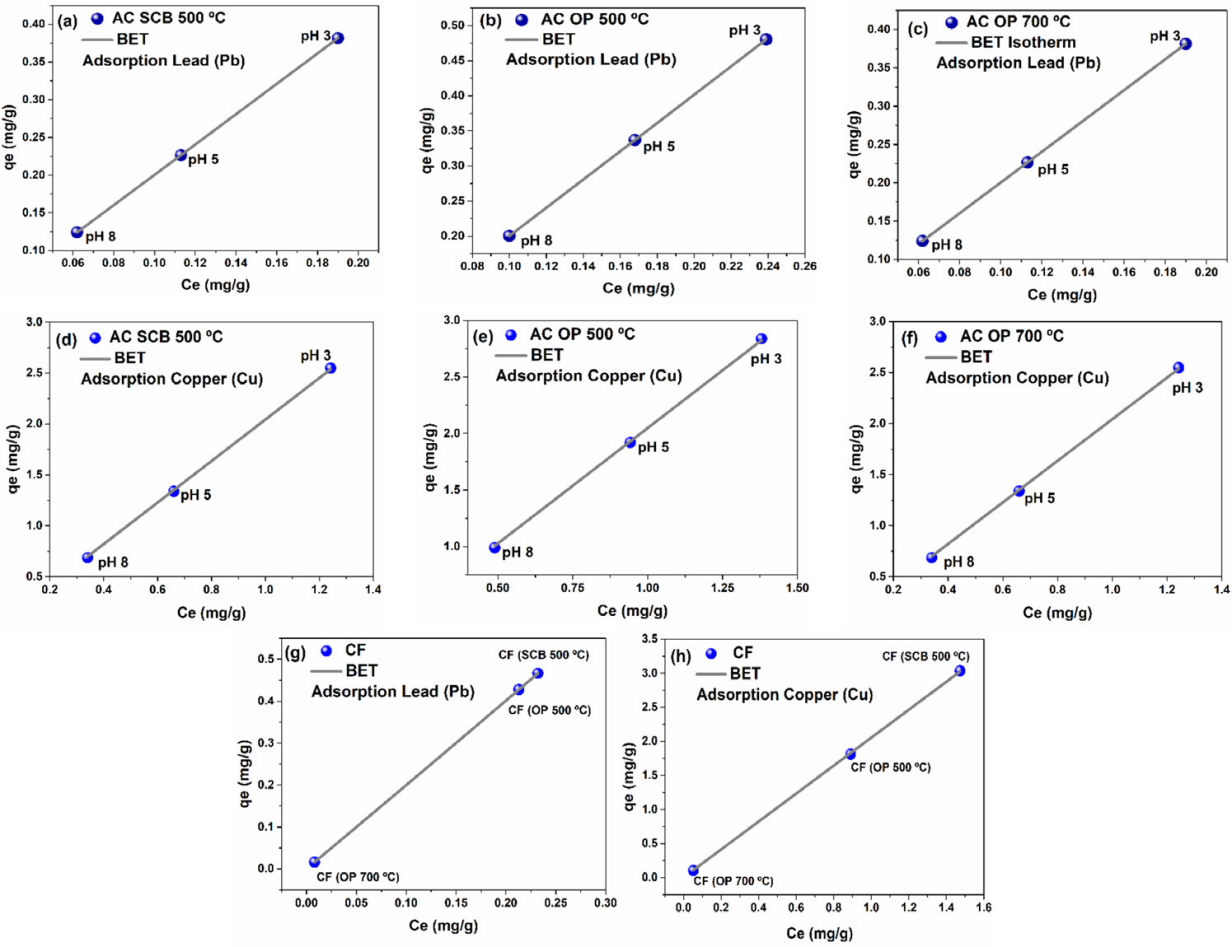
BET Isotherms of activated carbon and carbon foam after heavy metal removal.

Semi-quantitative analysis of activated carbons (SEM/EDS) was used to confirm the adsorption of heavy metals on the surface of activate carbons and the results are shown in Fig. 6 a–d. The data were fit to the BET equation to compare the modeling performed with Langmuir model, assuming that adsorption occurs in the monolayer adsorption, the results are shown in Fig. 7 a–h. Finally, the adsorption equilibrium parameters obtained from Freundlich-Langmuir isotherms using atomic absorption data can be seen in Table 1.

**Table 1:** Isotherm parameters for adsorption of Pb and Cu onto carbon adsorbents.

	Langmuir				Freundlich		
	Qmax (mg g^−1^)	KL (Lmg-1)	R^2^	RL	1/n	Kf (Lmg-1)	R^2^
	Adsorption Lead Pb						
ACs SCB 500 °C	97.649	0.020	0.999	0.492	0.0022	0.124	0.95
ACs OP 500 °C	106.32	0.018	0.999	0.526	0.0031	0.123	0.97
ACs OP 700 °C	97.64	0.020	0.999	0.492	0.0022	0.124	0.94
Carbon Foams	968.71	0.063	0.999	0.240	0.00129	0.124	0.99
	
	Adsorption Copper Cu						
ACs SCB 500 °C	754.142	0.002	0.999	0.880	0.0141	0.122	0.93
ACs OP 500 °C	706.824	0.002	0.999	0.872	0.0171	0.122	0.95
ACs OP 700 °C	754.142	0.002	0.999	0.880	0.0141	0.122	0.43
Carbon Foams	62.717	0.032	0.999	0.384	0.0077	0.1221	0.84

Thus, data from this research opens alternatives to propose orange peel and sugarcane bagasse to remove different types of contaminants in wastewater. Original and processed data can be found at https://data.mendeley.com/datasets/3gmbcyr5n7/2.

The data folder for the analysis of metal removal contains the graphs of activated carbon synthesized from orange peel and sugar cane bagasse at 500 and 700 °C, as well as the data sheets.

The BET isotherms folder contains the graphs derived from the analysis for the removal of copper and lead using activated carbon and carbon foams as a function of temperature and pH.

Particle size distribution analysis of hydrodynamic diameter during DLS measurements is presented in the form of histograms and distribution curves for activated carbon at 700 °C in the folder particle size analysis.

The folder labeled as pore size distribution contains the complete graphs of the activated carbon and carbon foam systems analyzed from both SCB and OP agroindustrial waste sources as a function of activation temperature. Source data are also reported.

Finally, an analysis of zeta potential (zeta potential folder) as a function of pH is presented for activated carbon and carbon foams at a temperature of 700 °C. Likewise, you will be able to find the source data obtained directly from the equipment used for the characterization.

## 2 Experimental Design, Materials and Methods

### 2.1 Preparation of Activated Carbons and Carbon Foams

Activated carbons were prepared from two agro-industrial wastes: orange peel (OP) and sugarcane bagasse (SCB), which were donated by Ingenio Pánuco S.A.P.I de C.V., and the OP was collected from the waste of a natural fruit juice. Initially, the samples were cleaned with distilled water and dried at 80 °C for 12 h. Subsequently, the raw materials were activated following the method previously reported [6], biomass degradation was carried out through the pyrolysis process under the following conditions: temperature at 400 °C under inert atmosphere (argon gas). In general, pyrolysis showed higher ash content, higher surface area development due to the loss of carbon components at high temperatures, and higher micropore volume, respectively [4]. The chemical activation consisted in the surface modification using an acid treatment; this activation, increases the specific area and generates a variety of pores, it adds chemical groups such as oxygen and nitrogen [5]. Two activation temperatures were used during the production of AC, 500 °C and 700 °C, and phosphoric acid (H_3_PO_4_ at 85 wt.%) was used as chemical reagent (Sigma Aldrich, 85% purity). Finally, the activated carbons were washed several times with distilled water until neutral pH was reached.

The replica method was used to obtain carbon foams with polyurethane foam as template and the activated carbon as precursor. The process was carried out in different steps: (i) solution preparation, (ii) structural conformation, (iii) drying and (iv) sintering [7]. Initially, 2 g of polyvinyl alcohol (PVA, 25 kDa, Fisher Scientific® 99% pure, 99% hydrolyzed), 20 mL of deionized water and 3 g of AC were mixed; the mixture was stirred at 60 °C until a homogenous mixture was obtained. Thereafter, the polyurethane foam with dimensions of 2.5 × 2.5 × 1.5 cm, was washed with deionized water for 10 min and dried at 60 °C for 3 h. Subsequently, the dry foam was put in contact with the mixture by immersion-compression until reaching an expansion within the suspension to achieve better infiltration. To do this, it was necessary to manually remove the excess material that remained on the sponge surface. The as-prepared carbon foams were dried in different stages; at 100 °C (1 h), thereafter temperature was increased up to 300 °C (1 h) and finally, a heat rate of 2 °C min^−1^ was used to sinter the samples at 800 °C (1 h). The sintering process was mainly applied to convert the fine particles into a coherent solid mass and increase the material porosity, as a result, the carbon foam showed micropore distribution and the template, organic materials and additives were further degraded. Carbon foams become brittle as the sintering temperature increases, which indicate that the density decreases as the temperature increases [8]. The adsorption capacity evaluation was carried out in synthetic water with a concentration of 50 mg g^−1^ of each metal (lead and copper). The adsorption capacity was evaluated at different pH's by regulating the solution at 3, 5 and 8.

### 2.2 Characterization Techniques

The particle size of the activated carbon was determined with a Litesizer 500 equipment (40 mW semiconductor laser of 658 nm). Activated carbons were dispersed in deionized water (Fisher Chemical HPLC) and the concentration was adjusted at 5 mg mL^−1^; then, sample was sonicated for 40 min. Subsequently, samples were diluted to different concentrations (3, 2 and 1 mg mL^−1^). The Zeta potential (ζ) determination was carried out in a Litesizer 500 Anton Paar equipment, with the following specifications: measuring range ≥ 1000 mV, mobility range 10^−11^ to 2 × 10^−7^ m^2^ V^−1^s^−1^, 1000 scans and at 25 °C.

The sample preparation consisted of approximately 0.05 g of carbon foam and activated carbon were added into 5 mL of deionized water; the sample was adjusted at different pH values from 2 to 8. The pH adjustment was performed with 0.01M HCl or 0.01 NaOH. Samples were sonicated for 40 min. The solution was transferred to a zeta potential cuvette for the measurement.

The pore size distributions of the activated carbon obtained at different temperature (500 °C and 700 °C) and of the carbon foam sintered at 800 °C were determined with Micromeritics Instrument TriStar II 3020 equipment. Approximately, 1 g of each sample was analyzed during 20 h, at 77.35 K in a nitrogen gas atmosphere. The pore size distribution was determined based on Barrett- Joyner- Halenda (BJH) method, which has been used to evaluate potential applications in biomass [9,10]. To determine the amount of heavy metals removed, samples were filtered using a 0.45 µm cellulose acetate syringe filter. The concentration of each metal was measured by atomic absorption spectrometry (AAS) according to the Mexican Standard NMX-AA-051-SCF1-2001. For the analysis of heavy metals, the flame method was used and calibration curves were made for each metal using high purity standards (Sigma Aldrich, 1000 mg L^−1^).

The removal efficiency (%) of carbon materials was calculated by the following equation [11].

(1) $$\text{Removal}\;\text{rate}=\frac{\text{Ci}-\text{Cf}}{\text{Ci}}X\;100$$

Where Ci and Cf are the initial and equilibrium concentration of the carbon material (mg L^−1^), respectively.

The semiquantitative analysis of activated carbon after the adsorption process was analyzed by scanning electron microscopy (SEM) using a JEOL JSM-6010LA apparatus, equipped with an elemental detector (EDS). The samples were fixed in a sample holder using graphite tape. Experimental absorption data were fitted to the Langmuir- Freundlich model by linear regression applying an iterative algorithm to obtain the maximum homogeneous biosorption capacity of heavy metal ions (Pb and Cu).

## Ethics Statements

The research does not involve using humans and animals as subjects. Also, the data were not collected from social media platform. The raw materials were donated by a sugar industry and the orange peel was collected as waste.

## CRediT authorship contribution statement

**Á.I. Licona-Aguilar:** Investigation, Methodology, Writing – original draft. **A.M. Torres-Huerta:** Validation, Resources, Writing – review & editing, Visualization, Formal analysis, Funding acquisition. **M.A. Domínguez-Crespo:** Visualization, Conceptualization, Methodology, Resources, Supervision, Funding acquisition. **D. Palma-Ramírez:** Visualization, Data curation, Writing – review & editing. **E. Conde-Barajas:** Methodology, Formal analysis, Resources. **M.X.L. Negrete-Rodríguez:** Methodology, Formal analysis, Resources. **A.E. Rodríguez-Salazar:** Writing – review & editing, Visualization. **D.S. García-Zaleta:** Data curation, Writing – review & editing.

## Declaration of Competing Interest

The authors declare that they have no known competing financial interests or personal relationships that could have appeared to influence the work reported in this paper.

## Data Availability

Data that support the use of agroindustrial wastes orange peel and sugarcane bagasse for the production of carbonaceous structures and their application in the metal ions remova (Original data) (Mendeley Data).
